# Serotonin Transporter Polymorphism Modulates N-Back Task Performance and fMRI BOLD Signal Intensity in Healthy Women

**DOI:** 10.1371/journal.pone.0030564

**Published:** 2012-01-23

**Authors:** Rune Jonassen, Tor Endestad, Alexander Neumeister, Kari Bente Foss Haug, Jens Petter Berg, Nils Inge Landrø

**Affiliations:** 1 Department of Psychology, Center for the Study of Human Cognition, Oslo, Norway; 2 Division of Psychiatry, Akershus University Hospital, Akershus, Norway; 3 Department of Psychiatry, Mount Sinai School of Medicine, New York, New York, United States of America; 4 Section for Research, Department of Medical Biochemistry, Oslo University Hospital, Ullevål, and Institute of Clinical Medicine, Oslo, Norway; Bellvitge Biomedical Research Institute-IDIBELL, Spain

## Abstract

**Context:**

Exploring intermediate phenotypes within the human brain's functional and structural circuitry is a promising approach to explain the relative contributions of genetics, complex behaviors and neural mechanisms in the development of major depressive disorder (MDD). The polymorphic region 5-HTTLPR in the serotonin transporter gene (SLC6A4) has been shown to modulate MDD risk, but the neural underpinnings are incompletely understood.

**Objective:**

37 right handed healthy women between 21 and 61 years of age were invited to participate in an fMRI modified n-back study. The functional polymorphism 5-HTTLPR located in the promoter region of the SLC6A4 gene was genotyped using polymerase chain reaction (PCR).

**Results:**

Short 5-HTTLPR allele carriers showed more blood-oxygen-level-dependent (BOLD) bilateral prefrontal cortex activation in the right [F(2, 30) = 4.8, η^2^ = .25, p = .026] and left [F(2, 30) = 4.1, η^2^ = .22, p = .015] inferior frontal gyrus pars triangularis with increasing n-back task difficulty relative to long 5-HTTLPR allele carriers. Short 5-HTTLPR allele carriers had inferior task performance on the most difficult n-back condition [F(2, 30) = 4.9, η^2^ = .26, p = .014].

**Conclusions:**

This activation pattern found in healthy at risk individuals resembles an activation pattern that is typically found in patients suffering from acute MDD. Altered function in these areas may reflect intermediate phenotypes and may help explain the increased risk of depression in short 5-HTTLPR allele carriers.

## Introduction

Altered lateral prefrontal cortex function is one of the most consistently reported findings in major depressive disorder (MDD) [1]. Both increased and decreased functioning in the medial- and lateral prefrontal cortex during working memory related tasks have been demonstrated in MDD relative to healthy control subjects [2], and these activation patterns appear to contribute to the development and progression of MDD. It is unclear, however, if these alterations represent a vulnerability factor that increases the risk for developing MDD, or rather develops as a consequence of MDD. The serotonin transporter polymorphism (5-HTTLPR), with a variable number of DNA sequence repeats (short and long alleles) located in the regulatory region of the gene, has repeatedly been shown to moderate MDD risk [3]. Several studies have demonstrated that short 5-HTTLPR carriers, particularly women [4], have increased vulnerability for the development of MDD in the context of stressful life events [5]. Cognitive control is a key process in an integrated cognitive-biological model of depression [6], but 5-HTTLPR influence on this top-down part of the system has not been systematically investigated. This is in contrast to a number of studies on the limbic system based bottom-up pathway. In particular, the amygdala's role in the perception of emotional valence has led to a series of studies to determine the role of the 5-HTTLPR in processing emotionally salient information in MDD [7]. Short 5-HTTLPR carriers have shown highly significant reduction of amygdala-anterior cingulate cortex connectivity in comparison to homozygote long 5-HTTLPR carriers [8]. Short 5-HTTLPR carriers have also shown more functional coupling between the amygdala and the ventromedial prefrontal cortex, compared to long 5-HTTLPR carriers [9]. There is also evidence of 5-HTTLPR-dependent structural variability in the MDD circuit that provides cognitive control of emotion (11). However, functional imaging studies on cognitive control are sparse. Cognitive control of emotion plays an important role in emotion downregulation when emotion activation is no longer adaptive [10]. Therefore, individual differences in the ability to perform emotion downregulation may contribute in an important way to the risk for developing MDD.

Herein, we determine intermediate endophenotypes in a circuit that has been consistently implicated in MDD [11] in healthy women at increased risk for developing MDD [4]. We used fMRI and an n-back task to unmask altered brain function in healthy women who were grouped by 5-HTTLPR genotypes. We tested the hypothesis that short 5-HTTLPR allele carriers, but not long 5-HTTLPR carriers may be normal under resting conditions but show altered brain function while performing the n-back task as expressed by elevated activation within the lateral prefrontal cortex. Subregions within the ventrolateral prefrontal cortex were predefined based on its role in conscious emotion regulation [12], [13]. The activation pattern would be similar to what has been shown in individuals during a MDD episode [2]. We also predicted that short 5-HTTLPR carriers would have weaker performance on the n-back task and that performancewill be inversely associated with lateral PFC activation.

## Material and Methods

### Ethics Statement

All data, including blood samples, were collected, stored and treated according to the principles expressed in the Declaration of Helsinki [14]. All participants provided written informed consent. The project was approved by the Regional Ethics Committee for Medical Research, North Norway.

### Participants

37 healthy, women free from drugs were recruited at the Center for the Study of Human Cognition, UiO to participate in an fMRI study and an n-back task. All participants underwent medical and psychiatric evaluations including the Diagnostic Interview for Genetic Studies [15], the Structural Clinical Interview for DSM-IV, Axis I and II disorders (SCID I and SCID II). Depression and anxiety symptoms were assessed using the Beck Depression Inventory (BDI) and the Beck Anxiety Inventory (BAI), respectively. The SCID interviews were collected and recorded by trained clinicians and were subjected to consensus diagnoses. Education level was classified by means of the International Standard Classification of Education [16]. General cognitive functioning was estimated from scaled scores from two subtests of the WAIS-III, Picture Completion and Similarities [17].

### Genotyping

Genotyping the biallelic 5-HTTLPR polymorphism, located in the regulatory region of the serotonin transporter gene (SLC6A4), was performed essentially as described in detail elsewhere [18]. A real-time fluorescence LightCycler instrument was used to amplify genomic DNA by polymerase chain reaction (PCR) in a final volume of 20 ul using LightCycler Faststart DNA SYBR Green kit (Roche cat no 12239264001) with specific primers (0.5 uM) [19] generating a long (L) 419 base pair (bp) or a short (S) 375 bp PCR product. Differences in product length depend on the variable number of a 22 bp tandem repeat (VNTR) sequence in the promoter region. Cycle conditions were initiated by 10 min denaturation (95°C) followed by 45 cycles at 95°C (10 s), 66°C (10 s) and 72°C (10 s). Based on the identification of a single nucleotide polymorphism (SNP) within the long variant, suggestions have been put forward that this is a triallelic functional polymorphism [20]. For the detection of the additional A>G SNP (rs25531), the PCR fragments were digested with 1 U MspI restriction enzyme (New England Biolabs, Beverly, Massachusetts) for 2 hour at 37°C. The PCR fragments contain two obligatory MspI sites, whereas the A>G substitution creates an additional MspI site. The PCR reaction followed by restriction digestion and gel electrophoreses provides classification of the S, L_A_ and L_G_ alleles. The triallelic classification was then reclassified into a biallelic functional model, based on the 5-HTTLPR-directed level of transcriptional activity of the transporter gene as follows: L_G_/S, L_G_/L_G_ and S/S genotypes were classified as S/S (low leveled RNA transcription); L_A_/S and L_A_/L_G_ genotypes were classified as L/S (intermediate leveled); and L_A_/L_A_ genotype was classified as L/L (high leveled). The three groups were analyzed separately without dichotomized genotype factors.

### fMRI acquisition and analysis

**Figure 1:**
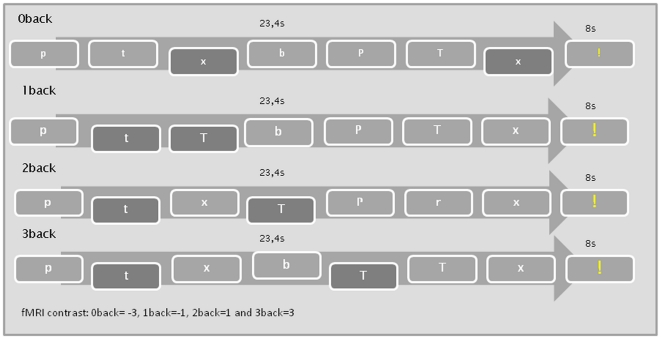
The fMRI modified n-back procedure. The n-back conditions were contrasted in a parametric block design.

In a working memory functional MRI paradigm, participants were instructed to monitor a series of stimuli and to respond whenever a stimulus was presented that was the same as the one presented n- trials previously. The increase in cognitive load is based on the parametric increase between the different n-backs. The paradigm was constructed in E-prime 2.0 studio software. The stimuli were a series of 2 times 12 small and large centred letters in 16 randomized blocks. Stimulus duration time was set to 300 ms with inter-stimulus interval fixation points of 1650 ms. Four types of stimulus procedures were randomized, two types containing 2 n-backs and two types containing 4 n-backs, giving a total of 48 events and 144 non events in each run (64/128 in the 0-back condition). Each of the 16 series had a total duration of 23,4 s. An 8000 ms resting condition was presented between series in the form of a centred exclamation point. Behavioural measures for group comparison were accuracy and reaction time. Outcome measures used in the fMRI analysis were onset time and duration in the n-back series compared to the 8000 ms resting conditions in a block related design (Figure 1).

**Figure 2:**
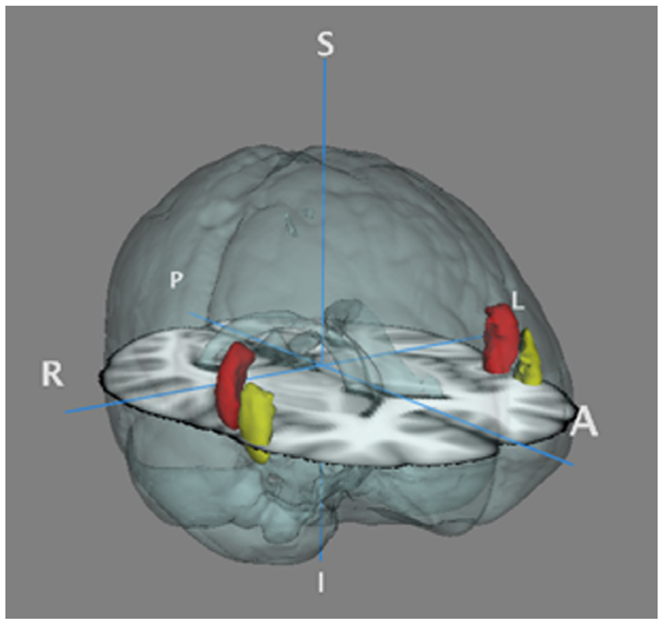
The two regions of interest masked based on the Harvard-Oxford Cortical atlas. All ROIs' intensity thresholds were set to 50–100 based on the probability maps for each label. Red = inferior frontal gyrus, pars opercularis. Yellow = inferior frontal gyrus, pars triangularis.

BOLD imaging data were acquired on a Philips Archieva 3T MR scanner using gradient echo EPI 34 transverse 3 mm slices (no gap), parallel to the AC- PC line. Repetition time (TR) = 2000 ms, slice echo time (TE) = 30 ms. Flip angle = 80%, field of view 240×240×102 mm. 3D structural images were 170 T1 weighted sagittal slices. FEAT, a part of the FSL software, was used in the model based fMRI analysis [21]. Data preprocessing included motion correction, first-level FILM GLM time series analysis and higher level FLAME Bayesian mixed effects analysis. The individual T1 weighted structural images were manually and individually prepared based on regions of interest (removing non brain regions such as neck, ears and nose), and scull stripped to remove non brain tissue. The linear registrations tool, FLIRT, was used for registration in 7 degrees of freedom from the individual functional images to T1 weighted images. Registrations from the individual T1 weighted images to standard MNI space were linear registrations [22]. The block related contrast was first included in a whole brain analysis for the whole sample to validate the fMRI modified n-back procedure and further included in ROI group (5-HTTLPR genotype) analyses based on the general linear model. ROIs were chosen a- priori and restricted to subdivisions within the prefrontal cortex that are involved in conscious downregulation of emotion [11]. The ROIs were defined based on the Harvard-Oxford Cortical Structure Atlas [23]. Two subregions of the VLPF, the inferior frontal gyrus, pars trinagualris and pars opercularis, were binary masked (threshold 50–100). The two subregions were further lateralized for the right and left hemisphere giving a total of 4 ROI's (Figure 2).

**Figure 3:**
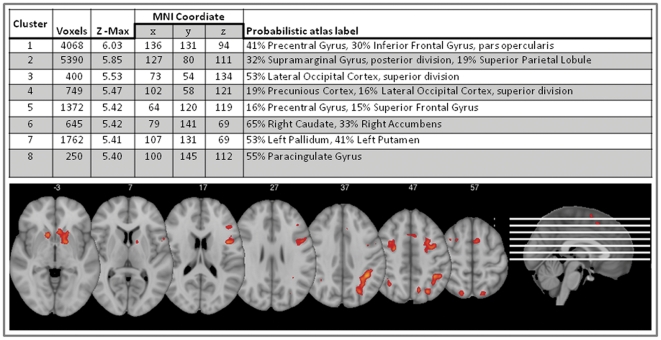
Location of BOLD contrast activation across the whole sample (n = 33). Z-threshold = 2.3 (p = .05). Clusters with Z-Max = >5.0 are displayed in the activation images.

Two way ANOVAs were conducted to explore potential group differences in age, education level, Beck Depression Inventory, Beck Anxiety Inventory, WAIS III; Similarities and Picture Completion, and the fMRI data processing was carried out using FEAT (FMRI Expert Analysis Tool) version 5.98, a part of FSL (FMRIB's Software Library). Z (Gaussianised T/F) statistics were determined by Z>2.3 and (corrected) cluster significance was thresholded using significance threshold of p = .05. Mid level analysis was carried out using a fixed effects model, by forcing the random effects variance to zero. Contrast parameter estimates (COPE's) were contrasted for each mask based on the parametric increase from the four n-back runs. Whole brain analysis was conducted to validate the n-back task. The BOLD activation across the whole sample resembled activation patterns reported from similar n-back designs [24]. The strongest activation was found within the lateral prefrontal cortex, the occipital cortex, the anterior cingulate cortex, and the basal ganglia. Location of the peak voxel was within the left lateral prefrontal cortex (Figure 3). Post hoc comparisons were conducted to explore the patterns of 5-HTTLPR x n-back condition.

Percentage signal change based on median COPE per non zero voxels was collected for each ROI. Two way ANOVAs were conducted using PASW Statistics 18 to explore potential effects of genotype within the 4 ROIs. Given the large age span, variance associated with age was accounted for by adding age as covariate in all analyzes. The behavioural measure of accuracy from events was reaction time corrected to prevent potential speed accuracy effects and separately analyzed for each n-back condition using two way ANOVA's.

## Results

Of the 37 women that were recruited into the study, one participant was excluded due to anxiety in the fMRI scanner and three participants were excluded based on low quality functional images. Data from 33 healthy women were used for the statistical analyses. The 5-HTTLPR genotypes did not differ in age, ISCED level, the two WAIS III scores, BDI or BAI (Table 1).

**Table 1:** Participant descriptive statistics.

	LL (n = 10)		LS (n = 11)		SS (n = 12)		Total (n = 33)	
	*Means±SD*	*Range*	*Mean±SD*	*Range*	*Means±SD*	*Range*	*Means±SD*	*Range*
**Age**	33.5±13.2	38	33.9±10.8	35	42.9±14.0	35	37.0±13.1	40
**ISCED level**	4.5±.8	2	4. 8±.8	3	5.1±.7	2	4.8±.8	3
**WAIS III PC**	13.0±2.3	8	14. 8±4.0	10	12. 7±3.0	10	13.5±3.2	10
**WAIS III SI**	11.1±3.0	10	10.5±2.3	9	11.2±3.4	13	11.0±2.9	13
**BDI**	2.0±2.4	8	1.3±2.1	7	1.5±2.5	9	1.6±2.3	9
**BAI**	1.7±1.3	4	.9±1.2	4	.7±.7	2	1.0±1.1	4

This table display mean, standard deviation and rage for 5-HTTLPR genotypes. LL = homozygous long carriers, LS = heterozygous carriers and SS = homozygous short carriers.

### Differences in BOLD contrast between 5-HTTLPR genotypes

**Figure 4:**
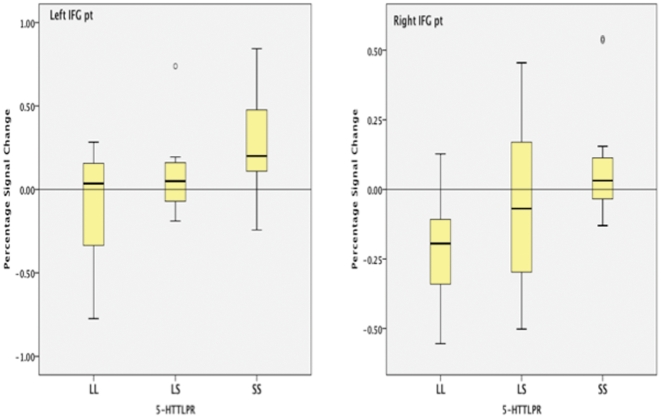
Box plots show the left and right Inferior Frontal Gyrus, pars triangularis interquartile range (50 percent of cases), median and range. Y-axis = percentage signal change, X-axis = 5-HTTLPR genotype (L = long, S = short).

The parametric contrast between n-back conditions revealed statistically significant age corrected main effects of 5-HTTLPR genotype for the right [F(2, 30) = 4.8, η^2^ = .25, p = .026] and left [F(2, 30) = 4.1, η^2^ = .22, p = .015] Inferior frontal gyrus, pars triangularis. No statistically significant effects were found within the left or right inferior frontal gyrus pars opercularis by applying the 5-HTTLPR genotype (Figure 4.).

**Figure 5:**
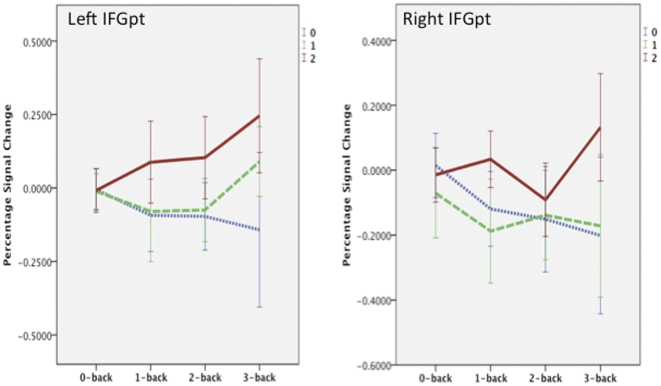
Percentage signal change per n-back condition for 5-HTTLPR genotypes. 0, 1, 2 = number of short alleles. Error bars show 95% confidence interval.

Post hoc comparision between n-back conditions showed a linear trend (5-HTTLPR x n-back) in both the left [F(2, 30) = 3.9, p = .031] and right [F(2, 30) = 3.7, p = .036] IFGpt. The ratio between activation and deactivation within ROIs was larger for the left hemisphere compared to the right hemisphere. Differnces between 5-HTTLPR are most pronounced in the most difficult condition in both hemispheres (Figure 5.).

### Differences Associated with fMRI Behavioral Measures

**Figure 6:**
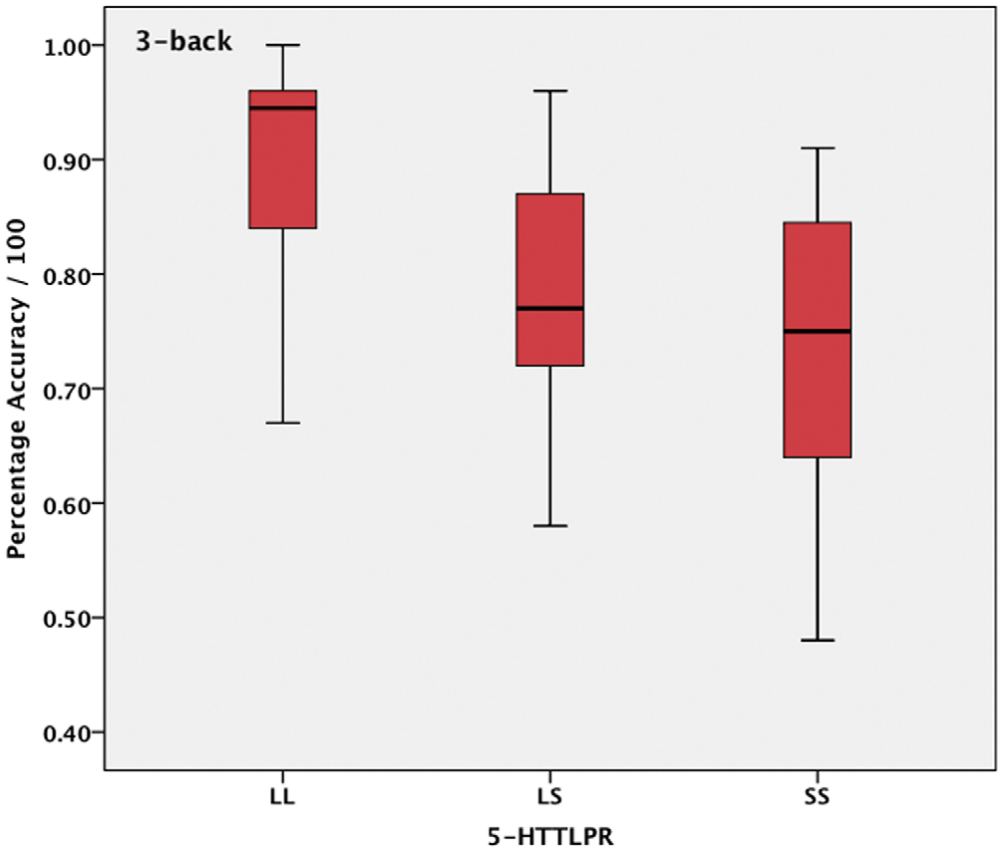
Boxplot shows the linear decrease in 3-back accuracy in short 5-HTTLPR carriers.

Age and reaction time corrected two way ANOVAs revealed a statistically significant difference between genotypes on accuracy for the 3-back condition [F(2, 30) = 4.9, η^2^ = .26, p = .014]. Applying a polynomial contrast revealed a statistically significant linear relationship [CE = −.123, p = .005] between number of short alleles and accuracy for the 3-back condition (Figure 6). There were no associations between genotype and n-back conditions for the 2-back (88±10.4) 1-back (96±8.6) and 0-back (98±3.4), which revealed gradually less inter subject variance compared to the 3-back condition (79±13.2).

## Discussion

The 5-HTTLPR polymorphism influences task performance and VLPFC activation pattern in healthy women during an n-back procedure. The fMRI modified n-back paradigm revealed a main effect of genotype within both the right and left VLPFC. Short 5-HTTLPR carriers showed higher percentage signal change within these subregions of the LPFC. Post hoc comparisons between n-back conditions revealed a linear trend, but the differences between genotypes were clearly most pronounced in the most difficult condition. Short 5-HTTLPR carriers compared to the long 5-HTTLPR carriers showed lesser reaction time corrected accuracy from events. This behavioral and neural pattern resembles impaired cognitive performance and exaggerated VLPFC activation in patients during a depressive episode [1].

The LPFC is important for maintaining and manipulating information in working memory. The VLPFC has shown robust activation in tasks that require cognitive control functions such as selection, comparison, and judgment of stimuli held in short- and long-term memory [24]. The increased activation within the VLPFC, combined with inferior performance on the n-back task in short 5-HTTLPR carriers suggests a relatively exaggerated cognitive load that may overwhelm an individual's ability to perform these cognitive control functions if needed, for example during stressful life events. Impaired function of these neural circuits are believed to contribute to the development of symptoms characteristic for MDD, such as systematic negative attention and recall biases [10]. The combined results suggest that the VLPFC responds in a modality-independent manner to a variety of explicit task demands. Functional imaging evidence indicates that left VLPFC is more active during conditions requiring goal-directed access to semantic knowledge [12]. Selecting among competing representations of task-appropriate knowledge is probably a substantial part of conscious emotion regulation [13]. Some authors have suggested that the well documented enhanced amygdala reactivity in short 5-HTTLPR carriers may lead to greater regulatory demands [25]. Lower functional connectivity between cortical regions that are critical to cognitive control of emotional responses to stimuli has been demonstrated [9], but does not explain what factors might contribute to these differences, neither how this may be linked to the serotonin system and the 5-HTTLPR. Individual differences in ones genetic make up may have equipped short 5-HTTLPR carriers with inferior cognitive control functioning compared to long 5-HTTLPR carriers.

Several limitations of this study should be noted. The decision to include only women in this study was based on the widely reported increased depression risk in women [26] and data showing a stronger association between short 5-HTTLPR and depression in the context of stressful life events seen in women [4]. Therefore, future studies need to determine whether our results are relevant for men. The sample consists of exclusively Norwegian participants and future studies should determine the generalizability of the results from this study involving other ethnic groups [27]. Finally, the limited sample size do not permit analyzes of gene×gene interactions. Studies on larger samples would also permit analyses of how the observed results relate to 5-HTTLPR variability reported in structural imaging studies, such as white matter integrity [28] and frontal gray matter volume [29].

The present study demonstrates impaired cognitive control and its neural correlate in healthy women carrying the short 5-HTTLPR allele. Our data may help explain the increased risk for depression in women carrying the short 5-HTTLPR allele.
